# Ongoing impacts of childhood-onset glomerular diseases during young adulthood

**DOI:** 10.1007/s00467-023-06250-z

**Published:** 2023-12-19

**Authors:** Keishiro Furuie, Shohei Kuraoka, Hideki Ban, Yuko Hidaka, Hiroko Nagata, Hiroshi Tamura, Koji Nagano, Tomoyasu Kawano, Akio Furuse, Hitoshi Nakazato, Kimitoshi Nakamura

**Affiliations:** 1 Department of Pediatrics, Faculty of Life Sciences, Kumamoto University, 1-1-1 Honjo, Kumamoto City, 860-8556 Japan; 2 Department of Pediatrics, Japanese Red Cross Kumamoto Hospital, Kumamoto City, Japan; 3 Department of Pediatrics, Kumamoto Chuo Hospital, Kumamoto City, Japan

**Keywords:** Childhood-onset glomerular disease, Distress, Transition, Higher education, Exercise restrictions

## Abstract

**Background:**

Childhood-onset glomerular disease often requires ongoing treatment and follow-up into adulthood. However, few studies have analyzed the associated impact and distress experienced by patients with this condition during the transition from childhood to adolescence and adulthood.

**Methods:**

At three facilities, we recruited patients who developed idiopathic nephrotic syndrome or IgA nephropathy during childhood and were at least 18 years old at the time of study entry. Among them, a questionnaire-based survey was administered to patients who consented to participate, and the results were analyzed in conjunction with clinical information.

**Results:**

Data from a total of 38 patients were analyzed. Of these patients, 15 had idiopathic nephrotic syndrome and 23 had IgA nephropathy. The age of transition from pediatrics to the adult medicine department was correlated with the number of recurrences. Many patients also reported being significantly affected by exercise restrictions and physical decline associated with their diseases and medications. Various impacts, including distress, affected decision-making regarding higher education, with patients engaging in higher education at a significantly higher rate compared with the regional average (66.7% vs. 46.9%, *p* = 0.028).

**Conclusion:**

We analyzed the impact of childhood-onset glomerular disease and distress during the transition period from pediatric to adult care. This study highlighted the significant impact of medications and exercise restrictions on patients’ decisions regarding higher education. Future prospective studies will be needed to examine patients’ distress in more detail and establish management approaches to enhance patient quality of life.

**Graphical abstract:**

**Figure:**
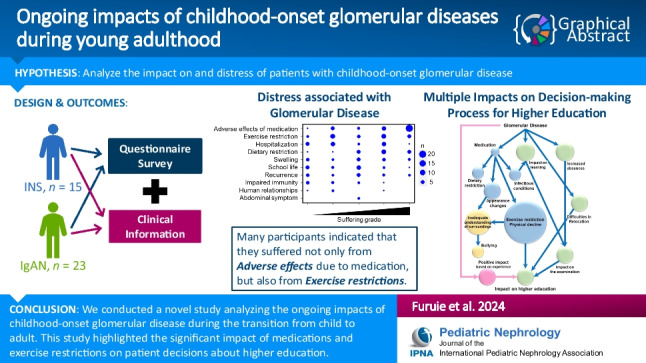
A higher resolution version of the Graphical abstract is available as Supplementary information

**Supplementary Information:**

The online version contains supplementary material available at 10.1007/s00467-023-06250-z.

## Introduction

Glomerular diseases refer to a group of kidney diseases characterized by inflammation and damage to the glomeruli that can affect individuals throughout their lives. In Japan, nephrotic syndrome, which is a typical glomerular disease, occurs at a higher frequency of 6.5 cases per 100,000 children per year compared with Western countries [1]. Approximately 90% of childhood-onset nephrotic syndromes are idiopathic nephrotic syndromes (INS), with histologic types including minimal change and focal glomerulosclerosis. Although the pathogenesis of INS remains unclear, it is believed to be related to immune function disorders, circulating factors, and anomalies in glomerular slit diaphragm proteins [2]. Minimal change nephrotic syndrome, the most common subtype, responds well to immunosuppressive therapy, with glucocorticoids used for initial remission induction [3]. However, patients with INS often experience repeated recurrences, leading to combination therapy with immunosuppressive agents, such as calcineurin inhibitors, and renin-angiotensin system inhibitors (RASI) to minimize long-term glucocorticoid adverse effects [3]. However, IgA nephropathy (IgAN) is primarily characterized by IgA-based immunoglobulin deposition in the glomerulus, leading to increased mesangial cell proliferation and substrate. IgAN is one of the most common types of chronic glomerulonephritis in Japan, with an estimated occurrence rate of 4.5–9.9 cases per 100,000 children per year [4, 5]. Proteinuria in IgAN does not generally reach nephrotic levels, and recurrence is less frequent compared to INS. Although clinical characteristics differ, the treatment approaches for IgAN are similar to those for INS, including glucocorticoids, immunosuppressive agents, and RASI [3].

Glomerular diseases including INS and IgAN progress to kidney failure when disease control is inadequate. Therefore, the impact on the lives of the children is extremely severe, often requiring long-term hospital visits and medications for treatments. Symptoms of glomerular disease, adverse effects of medications, and various lifestyle restrictions have been reported to negatively affect children’s quality of life [6–10]. These impacts are also known to affect the quality of life in adolescents and adults who develop glomerular disease in childhood [11, 12]. Although there are recommendations regarding the transition from pediatrics to adult medicine, there is insufficient evidence regarding the management of glomerular diseases during adolescence [13, 14]. Furthermore, few studies have focused on the transition to adolescence and adulthood in patients with childhood-onset glomerular disease. In this current study, we conducted a questionnaire survey to analyze the ongoing impacts quantitatively and qualitatively from childhood to adolescence and adulthood in patients with childhood-onset glomerular disease, as well as to investigate their actual experiences in school and social life.

## Methods

### Study participants

In the current study, we recruited patients who had developed INS or IgAN before the age of 15 and were at least 18 years old at the initiation of the study (October 1, 2022) from three facilities: Kumamoto University Hospital, Kumamoto Chuo Hospital, and Kumamoto Red Cross Hospital. We included patients who had been treated by pediatric nephrologists at these three facilities in Kumamoto Prefecture. The primary physicians who had been following the patients in pediatrics directly contacted them to confirm their willingness to participate in this study. Patients who agreed to participate provided written consent after receiving a detailed explanation of the purpose and content of the study.

### Data collection

Questionnaires were either mailed or hand-delivered to patients. To elucidate the various adverse effects associated with glomerular diseases, the questionnaires included the following information: age, height, weight, recent educational background, impact of the disease on schooling, current treatment, frequency of hospital visits, last hospitalization, concerns related to the disease, distress caused by the disease, and adverse effects of medications. We asked participants to select the top five most painful issues from the distress categories (Swelling, Abdominal symptom, Impaired immunity, Dietary restriction, Exercise restriction, Adverse effects of medication, Recurrence, Hospitalization, Human relationships, School life) and the adverse effect categories (Obesity/Moon face, Hypertrichosis, Feeling sick/Loss of energy, Gastritis/Stomach ulcer, Infection). The comments collected by open-ended questions were analyzed using the affinity diagram method. In the affinity diagram method, multiple evaluators categorized and illustrated each comment by group to derive relevance. Clinical information at the time of onset, treatment details, and the course of the disease were then collected by the primary physician from past medical records. The current estimated glomerular filtration rate (eGFR) was calculated by using the modified isotope dilution mass spectrometry-traceable 4-variable Modification of Diet in Renal Disease Study equation with the Japanese coefficient [15].

### Statistical analysis

Data such as physical examination, eGFR, duration of pediatric follow-up, and age at the end of pediatric follow-up are presented as mean with a 95% confidence interval (95% CI). To assess factors that could affect transition age and final height, multiple regression analysis was performed using the following variables: parameters at onset (INS or IgAN, age, sex, the urinary protein creatinine ratio, serum albumin, serum creatinine, hematuria, gross hematuria), total recurrence, treatment history (prednisolone, methylprednisolone pulse, mizoribin, cyclophosphamide, azathioprine, cyclosporin, mycophenolate mofetil, tacrolimus, rituximab, saireito, angiotensin-converting enzyme inhibitor, angiotensin 2 receptor blocker, warfarin, dipyridamole, omega-3 fatty acids, tonsillectomy). Additionally, Pearson’s chi-squared test was performed to compare the rate of higher education between the participants of this study and the regional population in Kumamoto Prefecture. All statistical analyses were performed using R version 4.1.3 (R Foundation for Statistical Computing, Vienna, Austria. http://www.R-project.org/). Differences with *p* values < 0.05 were considered to be statistically significant.

### KJ method

To analyze the qualitative data collected from the open-ended questions related to the impact on school life and higher education, we applied the KJ method, an affinity diagram method [16, 17]. After extracting various factors from comments, we combined duplicate or similar factors into groups, which we categorized as follows: Medication, Appearance changes, Infection condition, Dietary restriction, Exercise restriction, Physical declines, Increased absences, Impact on learning, Impact on examinations, Inadequate understanding of surroundings, Bullying, Impact on higher education, Difficulties in relocation, and Positive impact. Furthermore, we organized the visualization of relationships among these groups.

### Ethical issues

The current study was reviewed and approved by the Ethics Committees of Kumamoto University (No. 2573) at each participating institution. Participation of patients was permitted after obtaining written consent.

## Results

### Participant clinical characteristics

**Fig. 1:**
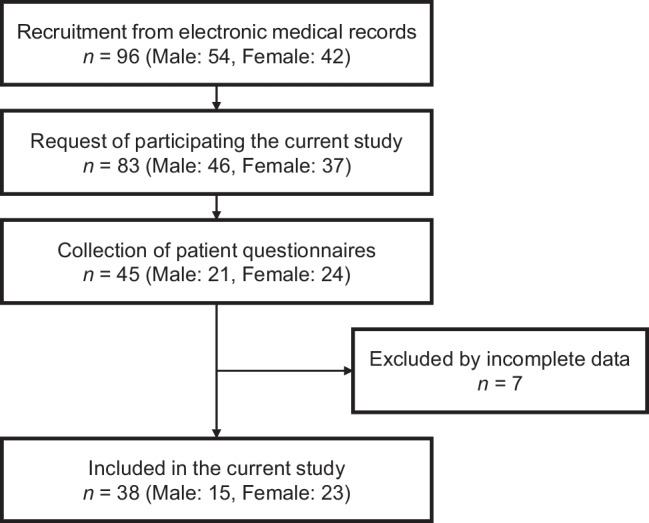
Recruitment diagram. A total of 96 participants were recruited for the current study using electronic medical records from three facilities. Finally, 15 INS patients and 23 IgAN patients were included in the analysis

Searching the electronic medical records for patients over 18 years old with INS or IgAN in childhood, the three facilities identified a combined total of 50 INS patients (30 males and 20 females) and 46 IgAN patients (24 males and 22 females). Among these patients, questionnaires were sent to 42 INS patients (25 males and 17 females) and 41 IgAN patients (21 males and 20 females) whose willingness to participate in the study was confirmed. Questionnaires were then collected from 22 INS patients (14 males and 8 females) and 23 IgAN patients (7 males and 16 females) patients (collection rate, 54.2%). After the exclusion of some patients because of missing clinical information, the final analysis included 15 INS patients and 23 IgAN patients (Fig. 1). Collection rates differed between genders, with more returns from female patients (male 45.7% vs. female 64.9%). There were no significant differences in collection rates based on the age of patients or the methods of distribution (mailing 58.3% vs. hand-delivering 58.8%).

The mean age of onset for INS and IgAN patients in this study was 9.6 and 11.4 years, respectively. This relatively high average age of onset for INS may have been caused by the limit of approximately 10 years on retrospective searches using electronic medical records. Otherwise, there were no other findings of note at the time of onset of disease in the participants in this study (Table 1). The responses to initial glucocorticoid treatment (2 mg/kg or 60 mg/m^2^ for 4 weeks) in INS patients were as follows: steroid-dependent, 8 (53.3%); frequent recurrence, 3 (20.0%); infrequent recurrence, 4 (26.7%). Among participants for whom a kidney biopsy was performed, nine INS patients exhibited minimal change, one INS patient had C1q nephropathy, and one INS patient had mesangial proliferative nephropathy. On the other hand, for IgAN, nine patients exhibited pathological changes in more than 80% of glomeruli and eight patients had crescent bodies in more than 30% of glomeruli.

**Table 1:** Summary of clinical characteristics

	INS *n* = 15		IgAN *n* = 23	
Profile at disease onset				
Age (95% CI)	9.6	(7.2–12.1)	11.4	(10.1–12.6)
Sex (%)	Male 8 Female 7	(53.3) (46.7)	Male 7 Female 16	(30.4) (69.6)
U-TP/U-Cr ratio (95% CI)	18.67	(9.57–27.78)	2.48	(0.92–4.04)
S-Alb (95% CI)	1.73	(1.30–2.17)	3.78	(3.44–4.12)
S-Cr (95% CI)	0.49	(0.41–0.57)	0.56	(0.44–0.68)
Hematuria (%)	6	(40.0)	22	(95.7)
Gross hematuria (%)	0	(0.0)	11	(47.8)
Recurrence (%)				
0	1	(6.7)	18	(78.3)
1–5 times	6	(40.0)	5	(21.7)
6–10 times	1	(6.7)	0	(0.0)
11–15 times	2	(13.3)	0	(0.0)
16–20 times	0	(0.0)	0	(0.0)
21–25 times	4	(26.7)	0	(0.0)
26–30 times	1	(6.7)	0	(0.0)
Treatment history				
Prednisolone (%)	15	(100.0)	22	(95.7)
Methylprednisolone pulse (%)	1	(6.7)	15	(65.2)
Mizoribin (%)	6	(40.0)	20	(87.0)
Cyclophosphamide (%)	1	(6.7)	0	(0.0)
Azathioprine (%)	0	(0.0)	2	(8.7)
Cyclosporin (%)	10	(66.7)	0	(0.0)
Mycophenolate mofetil (%)	4	(26.7)	0	(0.0)
Tacrolimus (%)	0	(0.0)	0	(0.0)
Rituximab (%)	7	(46.7)	0	(0.0)
Saireito (%)	1	(6.7)	0	(0.0)
ACEI (%)	6	(40.0)	17	(73.9)
ARB (%)	1	(6.7)	6	(26.1)
Warfarin (%)	0	(0.0)	10	(43.5)
Dipyridamole (%)	0	(0.0)	16	(69.6)
Omega-3 fatty acids (%)	0	(0.0)	0	(0.0)
Tonsillectomy (%)	0	(0.0)	12	(52.2)

Glucocorticoids were used as an initial treatment in all participants except one patient with IgAN (Table 1). Cyclosporine and mizoribine were the most common immunosuppressive agents used for INS, while mycophenolate mofetil (MMF) was less common because the use of MMF for INS was not covered by health insurance in Japan at that time. Rituximab, whose efficacy was confirmed by clinical trials in Japan, was also used in seven cases of INS [18]. In IgAN, however, methylprednisolone pulse therapy was administered in more than half of the cases. Mizoribine, which is recommended in Japan as a combination therapy, was the most commonly used immunosuppressive agent (Table 1) [19, 20]. Angiotensin-converting enzyme inhibitor (ACEI) and angiotensin 2 receptor blocker (ARB) were used in both INS and IgAN, but more frequently in IgAN.

### Current profiles and follow-up status

**Fig. 2:**
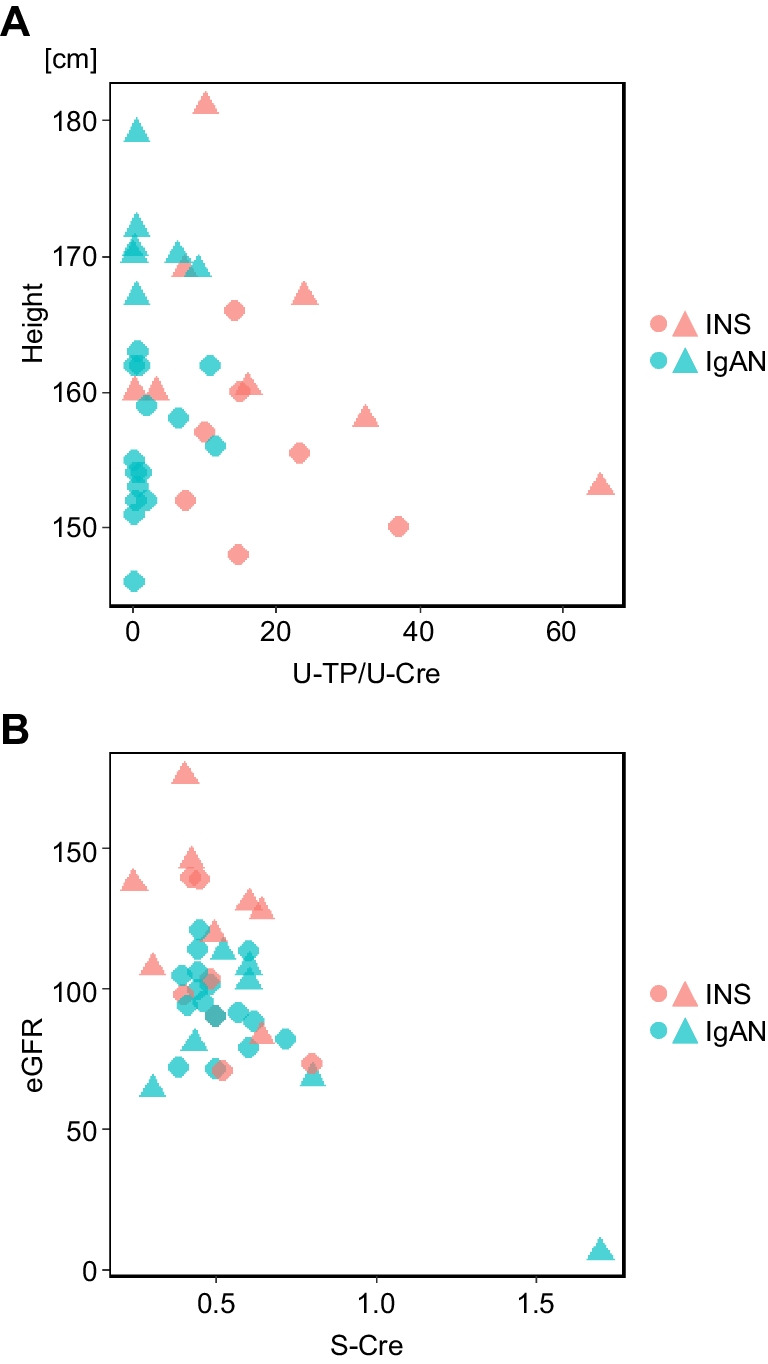
Correlation between final height and clinical information. **A** Scatter plots showing the correlation between final height and U-TP/U-Cr ratio at initial onset. **B** Scatter plots showing the correlation between final eGFR and serum Cr at initial onset. Each dot shape indicates gender. Circle, female; triangle, male

The mean age of the participants included in this analysis was 23.9 years for INS and 24.2 years for IgAN, respectively. There were no significant differences in height, weight, BMI, or age between the two diseases (Table 2). The eGFR was predominantly higher in the INS group than in the IgAN group, but only one IgAN patient who had undergone a kidney transplant had an eGFR less than 60 mL/min/1.73 m^2^. Multiple regression analysis was conducted on final height, considering disease and gender, and utilizing clinical findings at the time of initial onset, number of recurrences, and treatment history as variables. A negative correlation was observed in the urinary protein creatinine ratio (U-TP/U-Cr ratio) at the time of initial onset (*p* = 0.027) (Fig. 2A). Additionally, it was evident that the group receiving MMF treatment had a significantly shorter height (*p* = 0.010), while no significant difference was observed with methylprednisolone pulse therapy or other immunosuppressive agents (Supplemental Fig. 1). It should be noted that MMF administration for INS was not covered by health insurance in Japan, which likely led to patients resorting to MMF because of difficulties in maintaining remission with other immunosuppressive agents. No significant correlation was found between final height and the number of recurrences. In addition, we conducted a multiple regression analysis on eGFR with the same variables (Fig. 2B, Supplemental Table 1). Serum Cr at onset showed a negative correlation with eGFR (*p* = 0.003).

**Table 2:** Current profile and follow-up status

		INS		IgAN	
Age (95% CI)		23.9	(21.3–26.6)	24.2	(22.4–26.0)
Height (cm) (95% CI)	Male Female	163.5 155.5	(156.3–170.8) (149.8–161.2)	171.1 155.9	(167.5–174.6) (153.2–158.7)
Weight (kg) (95% CI)	Male Female	56.9 45.1	(47.9–65.9) (41.2–49.0)	67.7 52.4	(56.1–79.3) (46.1–58.8)
BMI (95% CI)	Male Female	21.2 18.7	(18.8–23.5) (17.0–20.4)	23.2 21.5	(19.0–27.4) (19.2–23.8)
eGFR (mL/min/1.73 m^2^) (95% CI)		115.9	(99.3–132.5)	89.6	(79.1–100.1)
Clinical turning point					
Pediatric follow-up continued (%) End of pediatric follow-up (%) Transition to other departments (%)		1 14 12	(6.7) (93.3) (80.0)	0 23 18	(0.0) (100.0) (78.3)
Pediatric follow-up duration (95% CI)		9.8	(6.3–13.3)	7.0	(4.8–9.2)
Age at end of pediatric follow-up (95% CI)		18.8	(16.5–21.1)	18.3	(17.3–19.3)

**Fig. 3:**
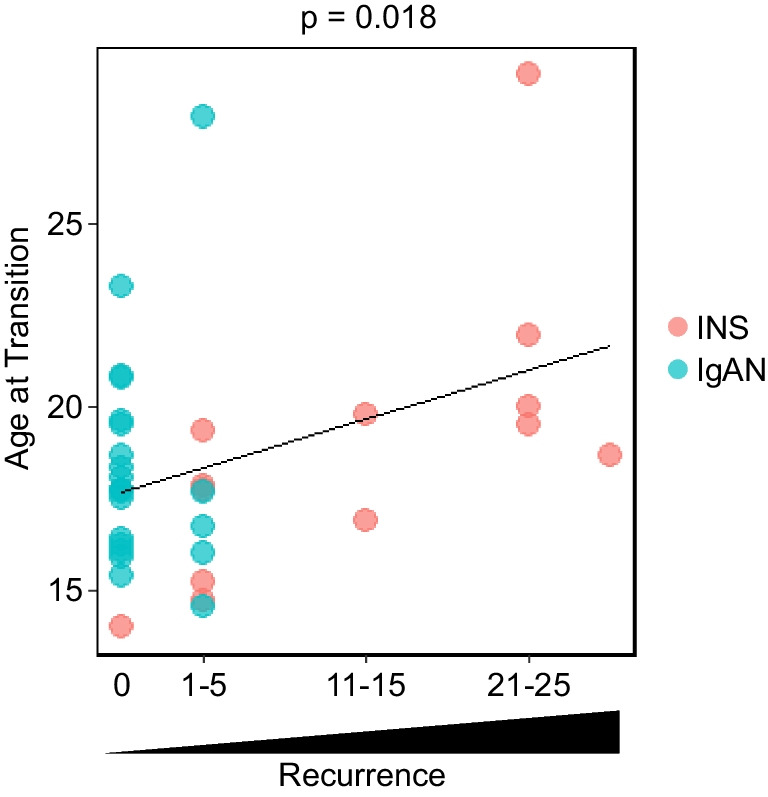
Correlation between age at transition and the number of recurrences. Participants were classified into seven groups according to the number of recurrences, and the association with age at transition was analyzed. Group 1, no recurrence; group 2, 1–5 times; group 3, 6–10 times; group 4, 11–15 times; group 5, 16–20 times; group 6, 21–25 times; group 7, 26–30 times

When the questionnaire was administered, follow-up in pediatrics had been completed in all but one case (Table 2). Of the 37 patients, seven had discontinued treatment and hospital visits, and 30 had been transitioned to the adult department for ongoing follow-up. The expected age for transitioning to adult services in our institution is typically 18 years. Specifically, patients with INS or IgAN transition at 18 years old, usually upon high school graduation. Nevertheless, they have the option to continue their follow-up until they reach age 20 or complete their higher education if they wish. The mean follow-up in pediatrics was 9.8 years for INS and 7.0 years for IgAN, and the mean age of transition from pediatrics was 18.8 years for INS and 18.3 years for IgAN, respectively. Hence, we analyzed the age of transition using disease, gender, clinical findings at the time of initial onset, and the number of recurrences as variables. No significant difference was found between INS and IgAN, but a high number of recurrences was detected as being positively correlated with age at transition (Fig. 3). Of the 30 patients who continued treatment, 22 were transitioned to the adult department because of their age, and the remaining eight were transitioned because of stable disease status. Most of the patients were transferred to nephrology, although one IgAN patient with kidney failure who received a kidney transplant was followed in the transplant surgery department. None of the cases utilized a transition program when transferring from pediatrics to adult departments.

### Distress from glomerular diseases and its impact on school life

**Fig. 4:**
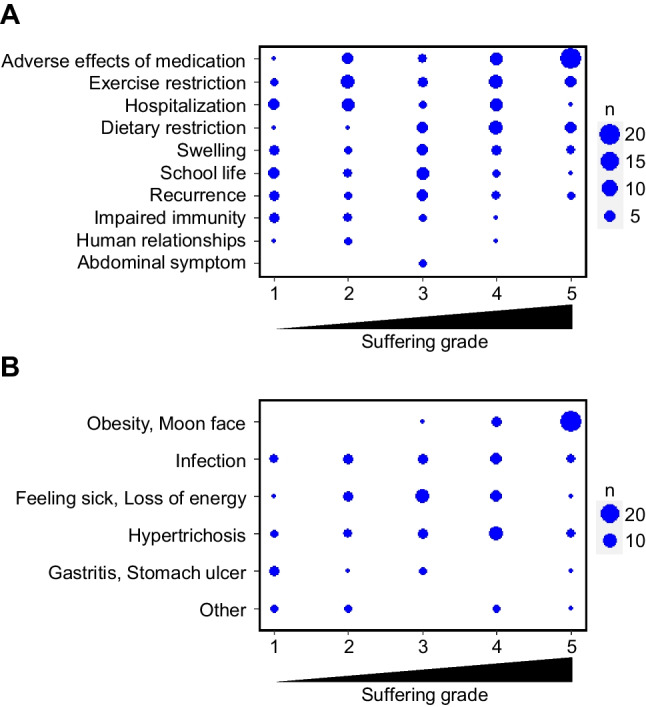
Distress associated with glomerular disease. Scatterplots depicting **A** distress caused by glomerular diseases and **B** distress caused by adverse effects of medications. The size of each mark represents the number of responses

We also administered a survey on distress caused by glomerular disease in our questionnaires. Adverse effects from medications were identified as the most painful type of distress caused by glomerular disease (Fig. 4A). Of these, obesity, moon face, and hypertrichosis, which are appearance-related issues, were reported to be the most painful (Fig. 4B). Other issues, which were frequently mentioned in questionnaires, included swelling, recurrence and hospitalization, and restricted exercise and diet. Although it was frequently mentioned as an adverse effect of medication, impaired immunity and the risk of infection did not seem to have a significant impact on overall distress. Interestingly, many participants indicated that the impact on their school life was distressing.

**Fig. 5:**
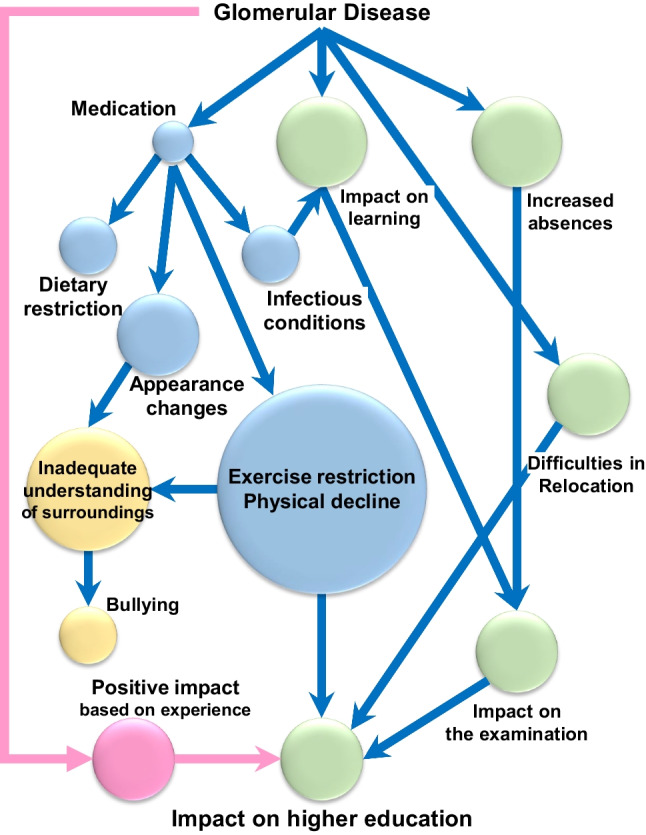
Affinity diagram of the impact of glomerular disease on school life and higher education. Blue bubbles: factors directly due to the disease or treatment; green bubbles: factors related to school life and learning; yellow bubbles: factors related to interpersonal relationships; pink bubbles: positive factors obtained due to the disease. The size of the bubbles indicates the number of answers obtained. Blue arrows, negative effect; pink arrows, positive effects

Next, we collected information on the impact on school life and higher education in the form of open-ended questions (Supplemental Table 2). The collected comments were then categorized and organized using the affinity diagram method, which is known as the KJ method [16, 17]. In this method, participant responses were initially extracted by factor. Similar factors were then aggregated, grouped, and given titles. Finally, we identified and visualized relationships, such as cause, effect, and modifiers, among the groups. The results obtained by the affinity diagram method are presented in Fig. 5. In categorizing the comments received from participants, negative impacts included the direct distress associated with glomerular disease and its treatment, the impact on school and learning, and stress in interpersonal relationships. The direct distress associated with glomerular disease and its treatment included infectious conditions and appearance changes caused by glucocorticoids and immunosuppressive agents. However, many participants (71.0%) mentioned the impacts of exercise restrictions and physical decline, which appeared to have a tremendous effect. Exercise restrictions and appearance changes affected patients’ formation of relationships with their surroundings, and a lack of sufficient understanding of patients’ symptoms and situation caused psychological distress. Additionally, apart from the impact of the disease on learning in school, increased absences because of hospital visits and hospitalization were frequently mentioned (23.7%). It was also indicated that they were unable to take the school examination itself due to their diseases. Furthermore, some participants indicated that it was challenging to continue seeing their primary facilities when they moved to distant locations. Conversely, positive impacts were also observed. Some participants’ comments suggested that their experience of having a glomerular disease and interactions with medical staff had a positive impact on their interest in medicine and their decision to pursue a medical career. These various impacts appeared to significantly contribute to some participants’ decision to pursue higher education. Importantly, the rate of engaging in higher education among participants in this study was significantly higher than the regional average in 2016 (https://www.e-stat.go.jp/dbview?sid=0000010105), which corresponds to the time at which participants were considering higher education (24 out of 36 [66.7%] vs. 7333 out of 15,622 [46.9%], *p* = 0.028). These findings suggest that various impacts associated with glomerular disease contribute to a high rate of advancement to higher education.

## Discussion

In the current study, we recruited participants with two diseases, INS and IgAN, to analyze the ongoing impacts of glomerular diseases during the transition from pediatric to adult care. We focused on INS and IgAN because these were reported to be the two most common glomerular diseases in a national survey in Japan [21]. The frequency of glomerular disease is higher in Japan compared with that in many other countries, and the diseases are diagnosed at an earlier stage because of an annual urinalysis screening program conducted in Japan from early childhood [1, 4, 5, 22]. While the two diseases differ in their clinical characteristics, such as the severity of proteinuria and the frequency of recurrences, their treatments share many similarities, including the use of glucocorticoids, immunosuppressive agents, and RASI. Because most of the participants recruited for the current study had INS or severe IgAN, glucocorticoids were used as the primary treatment in our sample. In the current study, when we analyzed the association between age at transition and clinical information, only the number of recurrences exhibited a significant correlation, not the variable of INS or IgAN. This suggests that regardless of INS or IgAN, a lower number of recurrences is associated with a younger age at transition. However, the recurrence behaviors for INS and IgAN are considerably dissimilar, and it would be hard to draw any definite conclusions from the number of analyses performed in this study. When analyzing medication factors affecting final height and transition age, we evaluated only the use of glucocorticoids and immunosuppressive agents. We found no significant differences, except for MMF. This result may be attributed to the fact that INS patients receiving MMF were refractory to treatment. However, the number of patients in our study was not large enough, and the combination of multiple medications made it challenging to address the effects of individual medications. In order to examine the effects of each drug in detail, future studies may need to analyze not only the dose and duration of medication but also the severity of the disease and patterns of combined therapy. Furthermore, more detailed information regarding the severity of proteinuria and the number of recurrences during the disease course would allow for a more accurate analysis. A well-designed prospective study may be preferable for this purpose.

Although it has been reported that childhood-onset glomerular disease affects patient quality of life, there are few reports on the actual impact during the transition period, and there are no established guidelines for the transition to adolescence and adulthood [6, 8–14, 23]. While some studies have analyzed the transition through questionnaires given to health care providers, studies that focus on the patients themselves, such as our study, are rare [24]. In the current study, we recruited patients aged 18 years and older, who may already be in the process of transitioning, to analyze the impact of glomerular diseases on life-stage shifts. This age group may have had an advantage over younger children in directly expressing their own experiences and feelings. Our questionnaire survey provided a detailed description of the impact and distress that patients with childhood-onset glomerular disease may experience during the transition, suggesting that glucocorticoid-based medications have the most significant impact and are at the root of their suffering. Glucocorticoids can cause not only immune function suppression, hypertension, and diabetes but also adverse effects with changes in appearance, such as moon face, obesity, and hypertrichosis [25–27]. In particular, increased bone resorption caused by glucocorticoids leads to secondary osteoporosis and increases the risk of fractures [28]. Consequently, exercise restrictions may be imposed on patients who show a decrease in bone mineral density during periodic follow-up. This exercise restriction, combined with glucocorticoid-induced muscle atrophy, causes a decline in physical health for patients. Interestingly, the survey revealed that many participants reported that exercise restriction and physical decline were distressing to them. Because of accumulating evidence that regular exercise in chronic kidney disease is effective for maintaining physical function and improving lifestyle without deterioration of kidney function, exercise restrictions for patients with chronic nephritis have been relaxed in Japan since 2012 [29, 30]. However, the impact of exercise restriction during childhood and adolescence still appears to be greater than expected. This suggests that health care providers need to take great care regarding decisions about imposing exercise restrictions on patients with glomerular diseases.

The current survey also suggests that the incidence of glomerular disease can influence the decision-making process for patients regarding higher education. Although there was a direct impact on patient learning, such as a decrease in study time and an increase in absences, some patients decided to continue their education to obtain specific qualifications, considering their physical decline. According to the responses to the questionnaire, patients believed that higher education would enable them to select a less physically demanding occupation. In addition, the positive impact of living with glomerular disease, serving as an incentive to pursue a medical career, may have also contributed to the significantly higher rate of higher education among participants in this study compared with the regional average. However, several limitations should be considered when interpreting these results. First, this study involved a small survey conducted in a single region, which might limit the generalizability of the findings. Second, the sample may have been biased; it is possible that a higher percentage of participants who selected higher education opted to participate in this study. Third, because there are many questions that require looking into the past, there may also be a recall bias. Therefore, more extensive research will be needed in the future to fully address and validate these findings. In addition, while the participants in this study were 18 years or older, future studies could include pediatric patients with glomerular disease to compare experiences during childhood with those during the transition period and beyond. In such cases, it will be necessary to carefully design a questionnaire that enables pediatric patients to adequately express their distress and feelings. Although not the focus of our study, it would also be necessary to analyze preparation for the transition, changes in feelings, and distress caused by the transition itself. We anticipate the accumulation of sustained research in the future.

## Conclusion

We conducted a study to analyze the ongoing impact of childhood-onset glomerular disease and distress on patients during the transition period from pediatric to adult care. This is the first study to focus on patients’ physical, psychological, and social distress, analyzed based on questionnaires answered by the patients themselves. Our study indicated that the distress caused by adverse effects of medication and exercise restrictions has a significant impact on patient decisions regarding higher education. In the future, more systematic prospective studies may be helpful for analyzing patient distress in detail and establishing management approaches that can improve patient quality of life.

### Supplementary Information

Below is the link to the electronic supplementary material.

Graphical abstract (PPTX 373 KB)

Supplementary file2 (DOCX 30 KB)

Supplementary file3 (XLSX 15 KB)

Supplementary file4 (PPTX 61 KB)

## Data Availability

A part of the datasets analyzed in the current study is not publicly available due to the risk of personal identification but is available from the corresponding author upon reasonable request.
