# Supplementary material for: Ongoing impacts of childhood-onset glomerular diseases during young adulthood
Source: Pediatr Nephrol. 2023 Dec 19;39(6):1791–9. doi: 10.1007/s00467-023-06250-z (PMC11026251; doi:10.1007/s00467-023-06250-z)
Supplement: Supplementary file 1 — Graphical abstract (PPTX 373 KB) [file 467_2023_6250_MOESM1_ESM.pptx]

## Slide 1
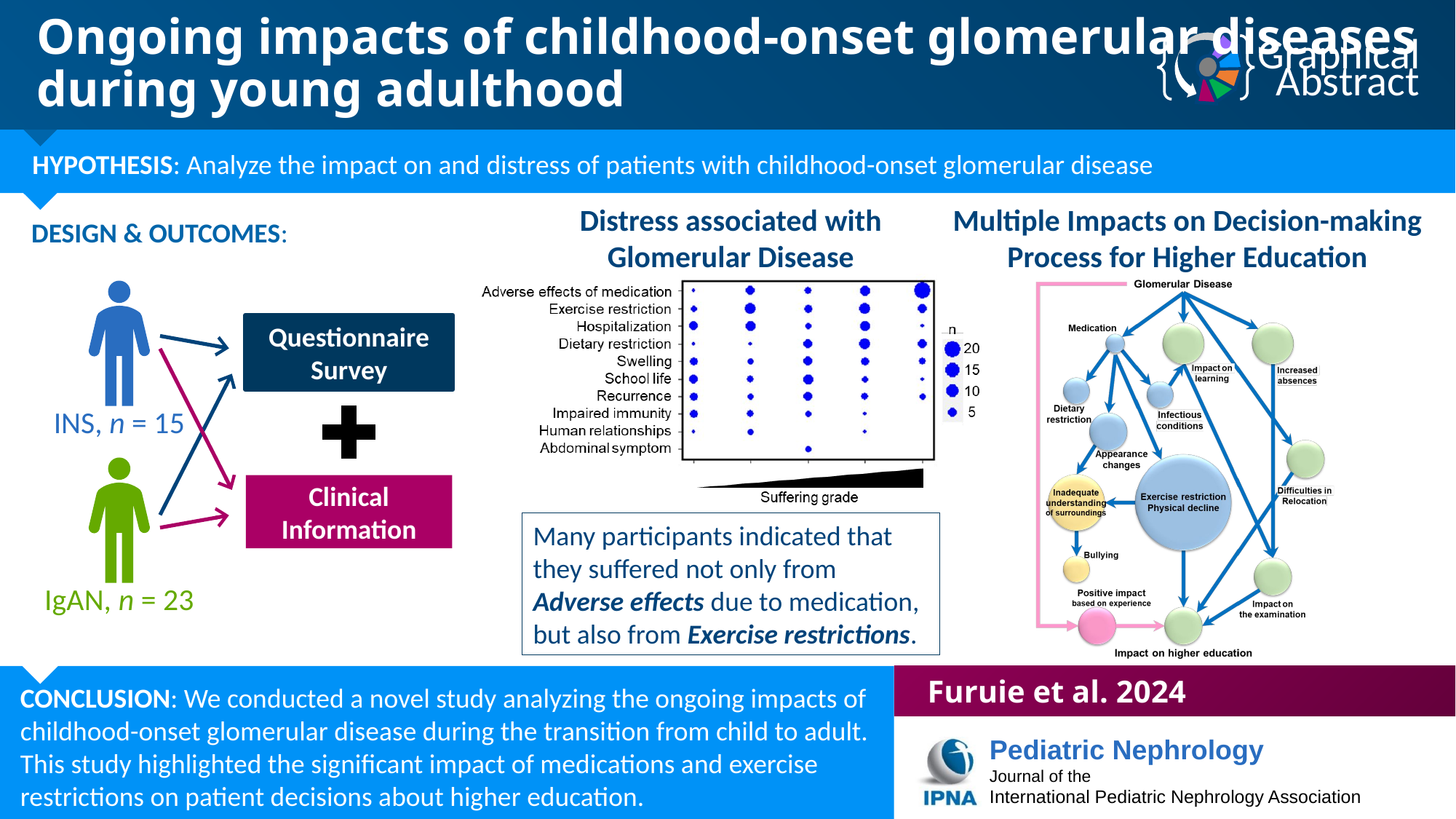

Ongoing impacts of childhood-onset glomerular diseases
during young adulthood
HYPOTHESIS: Analyze the impact on and distress of patients with childhood-onset glomerular disease
Distress associated with Glomerular Disease
Many participants indicated that they suffered not only from Adverse effects due to medication, but also from Exercise restrictions.
Multiple Impacts on Decision-making Process for Higher Education
DESIGN & OUTCOMES:
Questionnaire
Survey
Clinical
Information
INS, n = 15
IgAN, n = 23
Furuie et al. 2024
CONCLUSION: We conducted a novel study analyzing the ongoing impacts of childhood-onset glomerular disease during the transition from child to adult. This study highlighted the significant impact of medications and exercise restrictions on patient decisions about higher education.
