# Supplementary material for: Ongoing impacts of childhood-onset glomerular diseases during young adulthood
Source: Pediatr Nephrol. 2023 Dec 19;39(6):1791–9. doi: 10.1007/s00467-023-06250-z (PMC11026251; doi:10.1007/s00467-023-06250-z)
Supplement: Supplementary file 2 — Supplementary file2 (DOCX 30 KB) [file 467_2023_6250_MOESM2_ESM.docx]

**Supplemental Table 1: Relation of eGFR and treatment history**

|  | use group | | non-use group | |
| --- | --- | --- | --- | --- |
| **Treatment [mean (95%CI)]** |  |  |  |  |
| prednisolone | 100.8 | (91.1-110.6) | 68.0 | * |
| methylprednisolone pulse | 85.7 | (71.3-100.0) | 110.4 | (98.5-122.4) |
| mizoribin | 96.9 | (84.9-109.0) | 106.6 | (88.6-124.6) |
| cyclophosphamide | 175.6 | * | 98.0 | (89.0-106.9) |
| azathioprine | 92.3 | * | 100.4 | (90.3-110.6) |
| cyclosporin | 116.8 | (93.6-139.9) | 94.0 | (83.8-104.2) |
| mycophenolate mofetil | 136.9 | (88.7-185.2) | 95.7 | (86.4-104.9) |
| rituximab | 128.6 | (101.3-155.9) | 93.5 | (84.2-102.9) |
| saireito | 83.0 | * | 100.5 | (90.6-110.3) |
| ACEI | 100.3 | (89.3-111.2) | 99.6 | (79.9-119.3) |
| ARB | 112.5 | (83.1-141.8) | 97.2 | (86.7-107.7) |
| warfarin | 83.0 | (60.6-105.4) | 106.1 | (95.8-116.4) |
| dipyridamole | 85.5 | (71.5-99.4) | 110.6 | (98.5-122.7) |
| Tonsillectomy | 84.9 | (65.7-104.1) | 107.0 | (96.2-117.7) |

eGFR unit: mL/min/1.73m^2^, * n ≤ 2
