# Supplementary material for: Ongoing impacts of childhood-onset glomerular diseases during young adulthood
Source: Pediatr Nephrol. 2023 Dec 19;39(6):1791–9. doi: 10.1007/s00467-023-06250-z (PMC11026251; doi:10.1007/s00467-023-06250-z)
Supplement: Supplementary file 4 — Supplementary file4 (PPTX 61 KB) [file 467_2023_6250_MOESM4_ESM.pptx]

## Slide 1
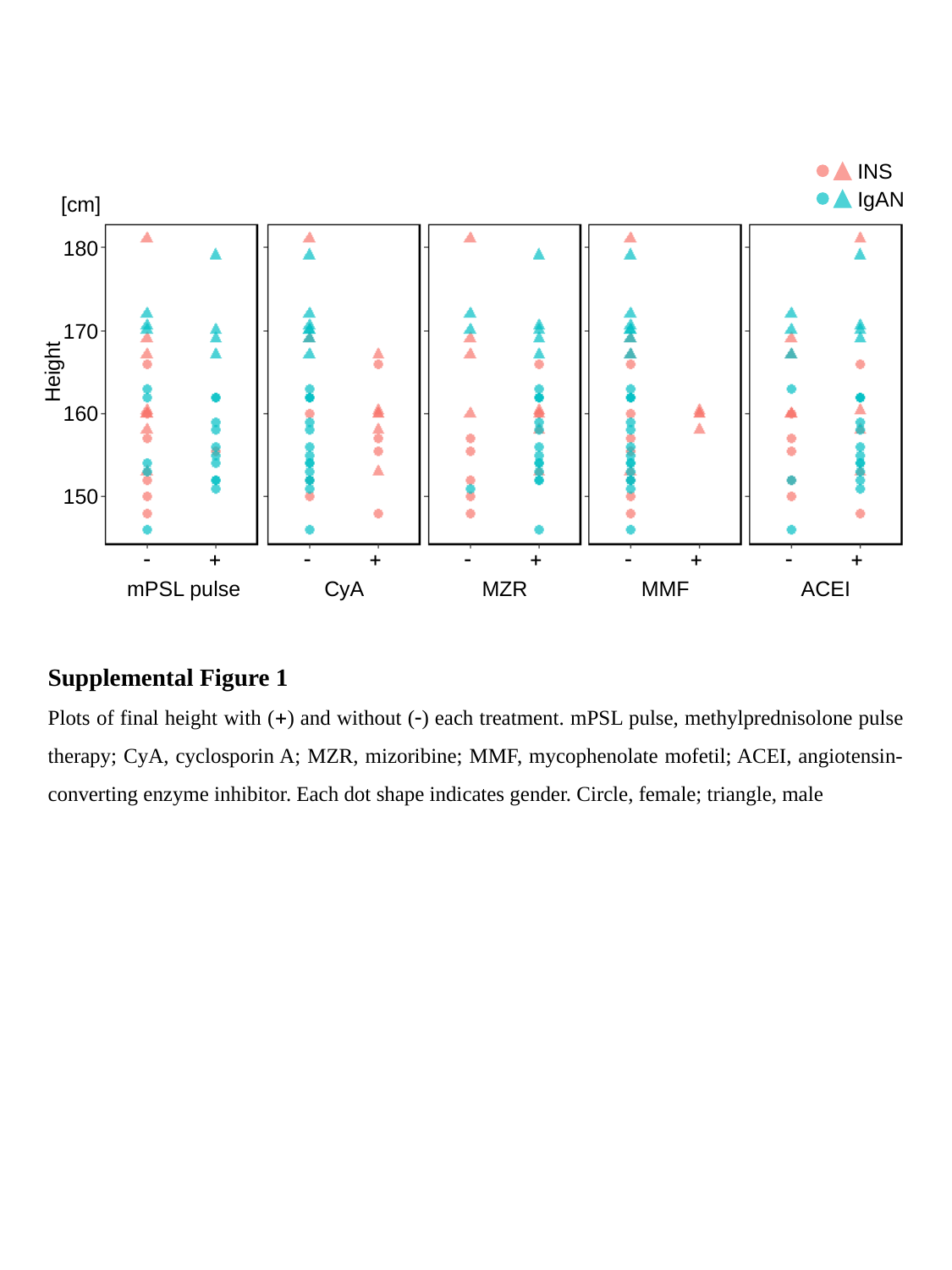

INS
IgAN
[cm]
180
170
Height
160
150
−
+
−
+
−
+
−
+
−
+
mPSL pulse
CyA
MZR
MMF
ACEI
Supplemental Figure 1
Plots of final height with (+) and without (−) each treatment. mPSL pulse, methylprednisolone pulse therapy; CyA, cyclosporin A; MZR, mizoribine; MMF, mycophenolate mofetil; ACEI, angiotensin-converting enzyme inhibitor. Each dot shape indicates gender. Circle, female; triangle, male
